# Hemodynamic Analysis of Pediatric Septic Shock and Cardiogenic Shock Using Transpulmonary Thermodilution

**DOI:** 10.1155/2017/3613475

**Published:** 2017-03-16

**Authors:** En-Pei Lee, Shao-Hsuan Hsia, Jainn-Jim Lin, Oi-Wa Chan, Jung Lee, Chia-Ying Lin, Han-Ping Wu

**Affiliations:** ^1^Division of Pediatric Critical Care Medicine, Department of Pediatrics, Chang Gung Memorial Hospital, Linko, Kweishan, Taoyuan, Taiwan; ^2^College of Medicine, Chang Gung University, Taoyuan, Taiwan; ^3^Division of Pediatric General Medicine, Department of Pediatrics, Chang Gung Memorial Hospital, Linko, Kweishan, Taoyuan, Taiwan

## Abstract

Septic shock and cardiogenic shock are the two most common types of shock in children admitted to pediatric intensive care units (PICUs). The aim of the study was to investigate which hemodynamic variables were associated with mortality in children with shock. We retrospectively analyzed 50 children with shock (37 septic shock cases and 13 cardiogenic shock cases) in the PICU and monitored their hemodynamics using transpulmonary thermodilution from 2003 to 2016. Clinical factors were analyzed between the patients with septic and cardiogenic shock. In addition, hemodynamic parameters associated with mortality were analyzed. The 28-day mortality was significantly higher in the septic group than in the cardiogenic group (*p* = 0.016). Initially, the parameters of cardiac output and cardiac contractility were higher in the septic group (*p* < 0.05) while the parameters of preload and afterload were all higher in the cardiogenic group (*p* < 0.05). Cardiac index was significantly lower in the nonsurvivors of cardiogenic shock at the time of initial admission and after the first 24 hours (both *p* < 0.05), while systemic vascular resistance index (SVRI) was significantly lower in the nonsurvivors of septic shock (*p* < 0.001). Therefore, during the first 24 hours after intensive care, SVRI and cardiac index are the most important hemodynamic parameters associated with mortality.

## 1. Introduction

Circulatory shock causes mortality in children and accounts for one-third of cases in intensive care units (ICUs) [1, 2]. Septic shock and cardiogenic shock are the two most common types accounting for three-fifth and one-fifth of the shock population, respectively, in ICUs [1, 2]. Some studies reported that the mortality rate was ~40 to 80% in septic shock and 60% in cardiogenic shock [3, 4]. Delay in the management and recognition of potential clinical symptoms/signs of compensated shock could lead to a high mortality rate [5]. Consequently, timely interventions to maintain an adequate tissue perfusion and oxygenation could significantly decrease the morbidity and mortality in children admitted to ICUs [6, 7]. Hemodynamic monitoring is essential for the diagnosis and therapeutic management of critically ill patients. Initially, physical examinations, vital signs, urine output, central venous pressure, and transthoracic echocardiography are often used to evaluate the preload and afterload status and cardiac functions in response to fluid resuscitation [8]. However, numerous studies recently demonstrated the inaccuracy of the methods of assessments for hemodynamic status compared to the objective hemodynamic parameter measurements [9–11]. Advanced hemodynamic monitoring may provide useful and precise data on preload, afterload, cardiac output (CO), cardiac contractility, and severity of pulmonary edema in patients with shock. In addition, assessing the severity of shock guided with an advanced hemodynamic monitoring may assist primary critical care physicians in treating patients and attribute a better clinical outcome.

Transpulmonary thermodilution, such as pulse index continuous CO (PiCCO), is a less invasive procedure (central venous and arterial catheters) and has been widely used in critically ill pediatric patients [12, 13]. Despite the frequent use of the PiCCO technique in pediatric patients, only few studies compared the hemodynamic parameters between the different types of shock and the chain of alternation between mortality and survival groups after treatment [14, 15]. In addition, there are insufficient data on what parameters are associated with mortality in critically ill pediatric patients. Therefore, the study aims to compare the parameters of septic and cardiogenic shock using the PiCCO system by analyzing the changes in hemodynamics in the mortality and survival groups. Moreover, we also identified the related parameters in predicting the survival and mortality in the critically ill pediatric patients with septic and cardiogenic shock.

## 2. Materials and Methods

### 2.1. Patient Population

This retrospective study of children aged 0 to 18 years presenting with shock to the pediatric ICU (PICU) was conducted in a tertiary medical center in Taiwan from 2003 to 2016. The PICU of our hospital was a tertiary ICU with 29 beds and hospitalized patients aged from 1 month to 18 years. The study criteria were uniformly applied to all patients screened in the study, making the study internally standardized based mainly on the international consensus conference, Paris, France, 2006 [16]. The types of shock categorized in mutually exclusive categories in the setting included septic and cardiogenic shock. The study was approved by the Institutional Review Board of Chang Gung Memorial Hospital.

### 2.2. Study Design

The critically ill children with hemodynamics monitoring via the PiCCO system (PiCCO, Pulsion Medical Systems, Munich, Germany) were included in this study. The transpulmonary thermodilution provided the following: (1) preload parameters: global end-diastolic volume index (GEDVI), intrathoracic blood volume index (ITBVI), and stroke volume variation (SVV); (2) cardiac parameters: CO, cardiac index (CI), and global ejection fraction (GEF); (3) afterload parameters: systemic vascular resistance index (SVRI); and (4) lung parameters: extravascular lung water index (EVLWI) and pulmonary vascular permeability index (PVPI). Information related to the cases of septic and cardiogenic shock included age; sex; cardiac characteristics, such as initial inotropic equivalent, heart rate (beats/min), and mean arterial pressure (MAP; mmHg); parameters of the PiCCO system; length of stay in the hospital and PICU; and mortality.

Two sets of measurements were analyzed and compared. Initial parameters were detected within 2 hours of enrollment after the PICU admission. Other data were obtained 24 hours after the critical care under the monitoring of the PiCCO system. Hemodynamic parameters were analyzed between the survivors and nonsurvivors in both the cardiogenic and septic groups. Moreover, we identified the predictors of mortality in the children with cardiogenic and septic shock. The primary outcome was the 28-day mortality rate in the PICU (death from any cause before day 28), and the secondary outcome was the ICU length of stay.

### 2.3. Measurement of PiCCO Parameters

Three consecutive cold boluses are required for each calibration to obtain the mean measurements [13]. Measurements were performed every 12 hours and whenever any hemodynamic deterioration developed. Data were recorded and exported to the computer using the PiCCO-VoLEF Data Acquisition software (version 6.0; Pulsion Medical Systems) combined with the PiCCO plus device (PC 8100 software version 5.1). The following formula was used:

(1) $$\begin{array}{c} \Delta SVRI=\frac{\left(24\text{-hour SVRI}-\text{baseline SVRI}\right)}{\text{baseline SVRI}}\times 100. \end{array}$$

### 2.4. Statistical Analysis

The Chi-square test, Fisher's exact test, Student's *t*-test, Mann–Whitney *U* test, and multivariate logistic regression analysis were used where appropriate. In the descriptive analysis, values were presented as means ± standard deviations (SDs). The difference between the groups was presented as 95% confidence intervals (CIs). For comparison of dichotomous variables between the groups, the Chi-square test or Fisher's exact test was used. Comparisons of continuous variables between the two groups were performed using the Mann–Whitney *U* test. Predicted probabilities of mortality and 95% CIs were calculated using the logistic regression model, and survival was analyzed using the Kaplan-Meier curve. Finally, the receiver operating characteristic (ROC) curve was applied to determine the ideal cut-off values for the hemodynamic parameters for mortality in shock. The test characteristics of the different cut-off values, including sensitivity, specificity, area under the ROC curve (AUC), positive likelihood ratio (LR^+^), and negative likelihood ratio (LR^−^), were also examined.

The AUC, calculated using the trapezoidal rule, was considered a standard measure for the diagnostic value of the parameter. An optimal test result had a value of 1.0, while a useless test result had a value of 0.5. The LR^+^ and LR^−^ were calculated for the best cut-off values. The criterion value indicated the value corresponding to the highest accuracy (minimal false negative and false positive results). Statistical significance was set at *p* < 0.05. All statistical analyses were performed using the SPSS software (version 22.0; SPSS Inc., Chicago, IL, USA).

## 3. Results

### 3.1. Demographics of the Children Implanted with the PiCCO Device

**Figure 1:**
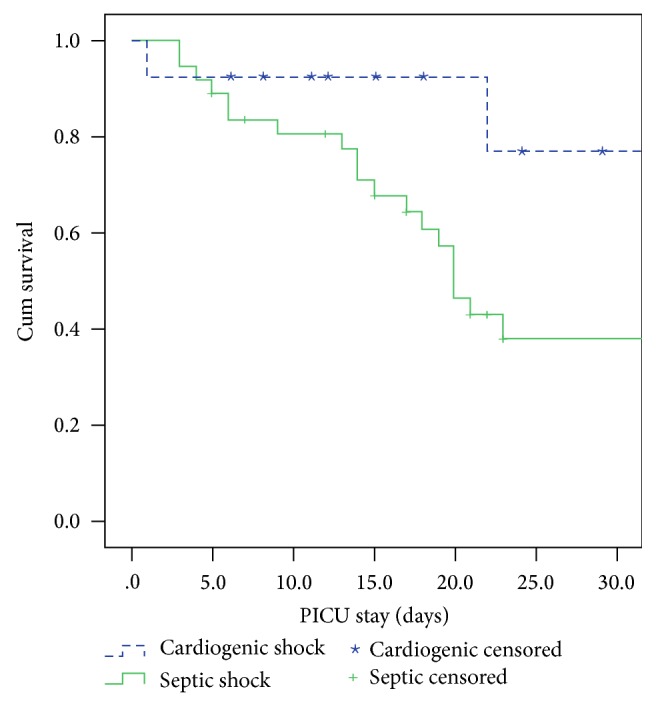
Survival rate analysis of children between septic and cardiogenic shock during the first 28 days of PICU stay (*p* < 0.05).

During the 13-year study period, 52 children with septic or cardiogenic shock monitored using the PiCCO system were gathered; however, two cases were excluded owing to insufficient data. Therefore, a total of 50 children were reenrolled in our study, with 30 male (60%) and 20 female (40%) patients (Table 1). There were 37 (74%) cases of septic shock and 13 (26%) cases of cardiogenic shock. The mean age was lower in the cardiogenic group (9.1 ± 6.1 years) than in the septic group (12.2 ± 4.5 years). The initial cardiac characteristics showed no significant difference between the two groups. However, the 28-day mortality rate was significantly higher in the septic group than in the cardiogenic group (59.5% versus 15.4%, *p* = 0.016) (Figure 1).

### 3.2. PiCCO Parameters at the Initial Admission and 24 Hours after PICU Admission

As shown in Table 1, the PiCCO parameters of CO and cardiac contractility, such as CI, GEF, and cardiac function index (CFI), were higher in the septic group than in the cardiogenic group (all *p* < 0.05). However, the parameters of preload and afterload, including the GEDVI, ITBVI, and SVRI, were higher in the cardiogenic group than in the septic group (*p* < 0.05). The factors between the survivors and nonsurvivors in both groups were identified and are shown in Tables 2 and 3. As shown in Table 2, the MAP was significantly lower in the nonsurvivors than in the survivors in the septic group at the time of PICU admission (*p* < 0.05). However, the CO and CI were significantly lower in the nonsurvivors in the cardiogenic group initially (both *p* < 0.05). The changes in the PiCCO parameters after treatment for 24 hours are presented in Table 3. The MAP was lower in the nonsurvivors than in the survivors in the septic group (*p* < 0.001). In addition, both the CO and CI were lower in the nonsurvivors than in the survivors in the cardiogenic group (both *p* < 0.05). However, notably, the SVRI was statistically and significantly lower in the nonsurvivors than in the survivors (901.08 ± 305.69 versus 1584.23 ± 429.63) in the septic group (*p* < 0.001).

### 3.3. Factors Associated with Mortality

**Figure 2:**
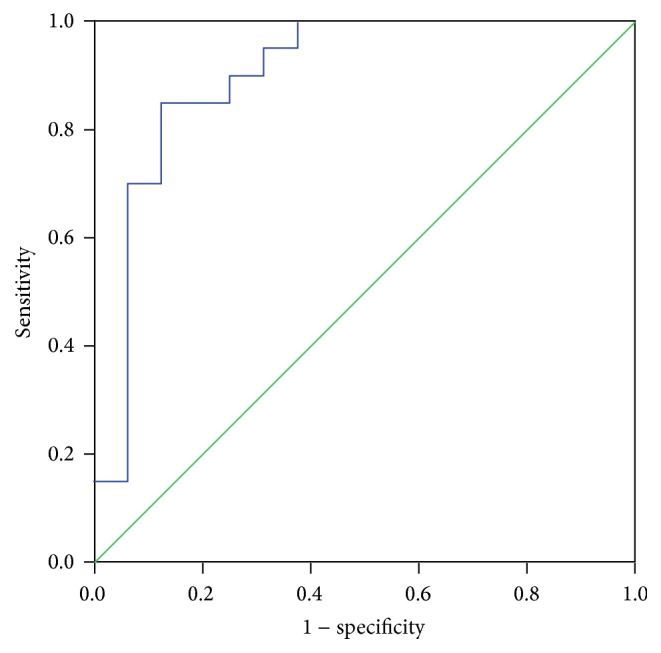
Receiver operator characteristic analysis for SVR in predicting mortality in septic shock after the 24 hours of admission to the PICU.

The results of the multivariate logistic regression analysis showed that SVRI was an independent predictor of mortality after the 24-hour critical care in the PICU in the septic group (odds ratio [OR], 0.995; 95% CI, 0.992–0.998, and *p* = 0.003). Based on the ROC analysis of SVRI in predicting the survivors in the septic group, the AUC was 0.9 (95% CI, 0.786–1, *p* < 0.001) (Figure 2). The cut-off values of SVRI in the septic group are shown in Table 4. We identified SVRI of 1167 dyn*∗*s*∗*cm^−5^*∗*m^2^ as the appropriate point to predict mortality. We also found that the change in SVRI (ΔSVRI) was negatively correlated with mortality (OR, 0.974; 95% CI, 0.952–0.997; *p* = 0.027).

## 4. Discussion

Shock is a major cause of morbidity and mortality in the PICU. In-hospital mortality rates of septic shock are high, ranging between 18% and 50% [1]. Mortality increases with the severity of sepsis. Hemodynamic monitoring is essential for the diagnosis and therapeutic management of critically ill patients. In the 13-year retrospective study, we found that SVRI was the most powerful predictor of the 28-day mortality in children with septic shock. There are few studies that demonstrate the importance of SVRI in adults with sepsis [17]; the present study is the first study to identify the importance of SVRI in predicting the mortality in children with septic shock. The SVRI of 1167 dyn*∗*s*∗*cm^−5^*∗*m^2^ during the first 24 hours after intensive care was the useful predictor of the 28-day mortality. In addition, we found that the change in SVRI (ΔSVRI) correlated with mortality negatively. In our study, the decreased CI in the children was an independent risk factor for mortality in the cardiogenic group, which was consistent with those of previous studies [18, 19].

A decreased SVRI indicates the expression of injuries in the endothelial layer; endothelial injuries are one of the important pathophysiologies of sepsis [20]. In sepsis, the injured endothelial cells could increase the secretions of reactive oxidants, lytic enzymes, prostacyclin, lipopolysaccharide, vasoactive substances, such as endothelin, platelet-derived growth factor, and the most important substance-overproduction of nitric oxide (NO) [20, 21]. Increasing NO synthesis by injured endothelial cells would damage the cerebral autonomic centers, which would further reduce the vascular reactivity to vasoconstrictors, causing a refractory hypotension [22, 23]. Another important factor causing hypotension in sepsis is the decreased compensatory secretion of vasopressin, which may be caused by impairing the baroreflex-mediated secretion [24]. Therefore, hypotension due to vasodilation, especially from endothelial injuries, may be the critical cause of circulatory malfunction in sepsis.

Although vasodilation induced by endothelial injuries may be the predictor of mortality in septic patients reported in some studies [20], the clinical application of SVRI has not been established in children. We estimated the association between the hemodynamic variables and clinical outcomes during the first 24 hours after intensive care because the therapeutic treatment during the early phase of shock could be crucial for survival [19, 25, 26]. The study demonstrated that the decreased value of SVRI after the 24-hour intensive care may serve as the early predictor of prognosis in children with sepsis, which is consistent with the results of a previous study in adults [17]. Several studies reported that the severity of pulmonary edema evaluated using the EVLWI and PVPI was the independent risk factor for mortality in sepsis [15, 18, 27]. However, although the nonsurvivors in our study had higher EVLWI and PVPI levels than that of the survivors, no significant difference was noted in the first 24 hours after the treatment. The difference may be that other studies analyzed the EVLWI at the maximum value and often developed 72 hours after intensive care, which is compatible with the clinical course of severe pulmonary edema commonly developing after 72 hours of intensive care [18, 27, 28].

On the other hand, CI was the independent risk factor for mortality in the pediatric cardiogenic shock in our study, and the results were consistent with those of previous studies in adults [19, 26, 29]. According to the pathophysiology, CI may be related to the base deficit. Our study observed that a decreased CI in the first 24 hours after intensive care could reflect the failure of hemodynamic interventions in nonsurvivors. Although only two nonsurvivors were included in our analysis, both cases had the lowest CIs among the cases of cardiogenic shock.

In conclusion, SVRI and CI are the most important hemodynamic parameters associated with the 28-day mortality in children with septic shock and cardiogenic shock, respectively, during the first 24 hours after intensive care. Most importantly, we determined the SVRI of 1167 dyn*∗*s*∗*cm^−5^*∗*m^2^ as the best appropriate predictor of mortality after 24-hour intensive care interventions.

## Figures and Tables

**Table 1:** Demographics of shock cases and initial PiCCO parameters.

Variables	Cardiogenic shock (*n* = 13)	Septic shock (*n* = 37)	*p* value
Age (years)	9.1 ± 6.1	12.2 ± 4.5	0.1
Gender			0.43
Male	9	21	
Female	4	16	
Cardiac characteristics			
inotropic equivalent	30.6 ± 30.3	46.4 ± 44.3	0.241
heart rate, beats/min	131.5 ± 33.2	138.2 ± 27.3	0.468
Mean arterial pressure, mm Hg	71.5 ± 15.1	69.4 ± 19.2	0.71
Outcomes			
Length of stay (days)	53.7 ± 85.7	34.8 ± 37.9	0.456
ICU stay (days)	28.1 ± 32.2	25.1 ± 32.4	0.779
Mortality	2	22	0.016
PiCCO parameters (Day 1)			
*Cardiac output*			
CO, L/min	2.68 ± 0.79	4.22 ± 1.65	<0.001
*Cardiac contractility*			
CI (L/min/m^2^)	2.84 ± 1.02	3.75 ± 1.08	0.011
GEF (%)	17.8 ± 8	27.95 ± 9.15	0.001
CFI (l/min)	5.73 ± 2.54	9.46 ± 2.76	<0.001
*Preload parameters*			
GEDVI (mL/m^2^)	519.11 ± 134.53	420.54 ± 118.01	0.017
ITBVI (mL/m^2^)	648.38 ± 168.38	525.23 ± 147.59	0.017
SVV (%)	13.84 ± 5.49	15 ± 6.5	0.568
*Afterload parameters*			
SVRI (dyn*∗*s*∗*cm^−5^*∗*m^2^)	1936.79 ± 802.41	1327.34 ± 705.48	0.013
*Lung parameters*			
EVLWI (mL/m^2^)	18.46 ± 12.01	14.8 ± 12.19	0.359
PVPI	3.99 ± 2.79	3.88 ± 2.53	0.898

ICU = intensive care unit; CO = cardiac output; CI = cardiac index; GEF = global ejection fraction; CFI = cardiac function index; GEDVI = global end-diastolic volume index; ITBVI = intrathoracic blood volume index; SVV = stroke volume variation; SVRI = systemic vascular resistance index; EVLWI = extravascular lung water index; PVPI = pulmonary vascular permeability index.

**Table 2:** Initial PiCCO parameters between survivors and nonsurvivors in children with cardiogenic shock and septic shock at the time of admission to the PICU.

Characteristics	Cardiogenic Shock			Septic shock		
	Death (*n* = 3)	Survival (*n* = 11)	*p* value	Death (*n* = 22)	Survival (*n* = 15)	*p* value
Age (years)	8.7 ± 10.89	9.15 ± 5.69	0.927	12.62 ± 4.3	11.53 ± 4.81	0.476
Gender			0.522			0.729
Male	1	8		13	8	
Female	1	3		9	7	
LOS in ICU	11.5 ± 14.85	31.01 ± 34.04	0.453	24.05 ± 38.43	26.73 ± 22.02	0.808
Cardiac characteristics						
inotropic equivalent	63.75 ± 76.91	24.55 ± 17.04	0.598	56.11 ± 54.33	32.17 ± 15.93	0.107
heart rate (beats/min)	121.75 ± 32.17	132.23 ± 34.63	0.673	137.83 ± 28.51	138.94 ± 26.38	0.906
Mean arterial pressure (mm Hg)	67.34 ± 26.4	72.3 ± 14.09	0.687	64.24 ± 17.73	76.87 ± 19.4	0.048
PiCCO parameters						
*Cardiac output*						
CO (L/min)	1.6 ± 0.06	2.88 ± 0.69	0.028	4.31 ± 1.59	4.09 ± 1.76	0.705
*Cardiac contractility*						
CI (L/min/m^2^)	1.34 ± 0.09	2.93 ± 0.98	0.049	3.68 ± 0.93	3.86 ± 1.29	0.626
GEF (%)	15.25 ± 4.59	18.27 ± 8.55	0.645	27.49 ± 10.26	28.56 ± 7.72	0.739
CFI (l/min)	4.48 ± 2.93	5.91 ± 2.58	0.584	9.01 ± 2.59	10.08 ± 2.95	0.263
*Preload parameters*						
GEDVI (mL/m^2^)	458.75 ± 51.97	530.08 ± 143.48	0.514	424.52 ± 107.12	415.23 ± 134.88	0.822
ITBVI (mL/m^2^)	572.41 ± 65.17	662.19 ± 179.54	0.512	530.18 ± 134.03	518.64 ± 168.63	0.823
SVV (%)	12.58 ± 0.12	14.07 ± 5.98	0.741	15.54 ± 7.23	14.21 ± 5.39	0.55
*Afterload parameters*						
SVRI, dyn*∗*s*∗*cm^−5^*∗*m^2^	1794.4 ± 219.08	1962.67 ± 873.53	0.798	1196.75 ± 509.52	1510.17 ± 901.11	0.193
*Lung parameters*						
EVLWI, mL/m^2^	18.16 ± 3.06	18.52 ± 13.18	0.97	15.34 ± 13.51	14.09 ± 10.58	0.769
PVPI	4.02 ± 2	3.98 ± 2.99	0.99	3.99 ± 2.84	3.74 ± 2.12	0.774

ICU = intensive care unit; CO = cardiac output; CI = cardiac index; GEF = global ejection fraction; CFI = cardiac function index; GEDVI = global end-diastolic volume index; ITBVI = intrathoracic blood volume index; SVV = stroke volume variation; SVRI = systemic vascular resistance index; EVLWI = extravascular lung water index; PVPI = pulmonary vascular permeability index.

**Table 3:** The PiCCO parameters between survivors and nonsurvivors after 24 hours of setting up the PiCCO.

Variables	Cardiogenic shock			Septic shock		
	Death (*n* = 3)	Survival (*n* = 11)	*p* value	Death (*n* = 22)	Survival (*n* = 15)	*p* value
Cardiac characteristics						
heart rate (beats/min)	120 ± 33.9	137.3 ± 39.2	0.572	139.18 ± 26.26	125.64 ± 32.45	0.169
Mean arterial pressure (mm Hg)	68.5 ± 23.3	79.9 ± 12	0.292	59.8 ± 14.84	84.13 ± 19.19	<0.001
PiCCO parameters						
*Cardiac output*						
CO (L/min)	1.53 ± 0.01	3.57 ± 0.97	< 0.001	5.01 ± 1.59	3.99 ± 1.55	0.062
*Cardiac contractility*						
CI (L/min/m^2^)	1.33 ± 0.11	3.59 ± 1.32	0.039	4.23 ± 0.92	3.77 ± 1.01	0.166
GEF (%)	15 ± 4.24	19.4 ± 7.56	0.451	30.93 ± 11.29	30.02 ± 11.46	0.812
CFI (l/min)	4.65 ± 2.76	6.56 ± 2.45	0.336	9.69 ± 3.34	9.09 ± 2.92	0.574
*Preload parameters*						
GEDVI (mL/m^2^)	452.5 ± 47.38	572.06 ± 106.39	0.157	457.82 ± 140.55	449.44 ± 156.64	0.867
ITBVI (mL/m^2^)	565 ± 59.39	714.82 ± 132.98	0.156	571.84 ± 175.74	561.3 ± 195.83	0.866
SVV (%)	11.25 ± 3.18	13.48 ± 5.72	0.61	15 ± 5.97	11.3 ± 5.87	0.068
*Afterload parameters*						
SVRI, (dyn*∗*s*∗*cm^−5^*∗*m^2^)	1742 ± 270.11	1664.48 ± 469.75	0.829	901.08 ± 305.69	1584.23 ± 429.63	<0.001
*Lung parameters*						
EVLWI (mL/m^2^)	16.5 ± 2.12	17 ± 11.63	0.954	16.5 ± 13.85	11.88 ± 6.53	0.229
PVPI	3.65 ± 1.63	3.06 ± 1.52	0.626	4.07 ± 2.8	3.09 ± 1.36	0.212

ICU = intensive care unit; CO = cardiac output; CI = cardiac index; GEF = global ejection fraction; CFI = cardiac function index; GEDVI = global end-diastolic volume index; ITBVI = intrathoracic blood volume index; SVV = stroke volume variation; SVRI = systemic vascular resistance index; EVLWI = extravascular lung water index; PVPI = pulmonary vascular permeability index.

**Table 4:** Predictive power of SVRI for different cut-off points in noncardiogenic group.

SVRI value	Sensitivity	Specificity	LR^+^	LR^−^	Youden index
533	0.15	1.0	—	0.85	0.15
591	0.15	0.938	2.4	0.907	0.0875
1093	0.7	0.937	11.2	0.32	0.6375
1115	0.7	0.875	5.6	0.347	0.575
1167^*∗*^	0.85	0.875	6.8	0.171	0.725
1351	0.85	0.75	3.4	0.2	0.6
1362	0.9	0.75	3.6	0.133	0.65
1371	0.9	0.687	2.88	0.145	0.5875
1394	0.95	0.687	3.04	0.073	0.6375
1460	0.95	0.625	2.533	0.08	0.575
1531	1.0	0.625	2.667	0	0.625

LR^+^: likelihood ratio for a positive test; LR^−^: likelihood ratio for a negative test;

*∗*: best cut-off point.

## Abbreviations

PICU: Pediatric intensive care unit
PiCCO: Pulse index continuous cardiac output
GEDVI: Global end-diastolic volume index
ITBVI: Intrathoracic blood volume index
SVV: Stroke volume variation
CO: Cardiac output
CI: Cardiac index
GEF: Global ejection fraction
SVRI: Systemic vascular resistance index
EVLWI: Extravascular lung water index
PVPI: Pulmonary vascular permeability index
LOS: Length of stay
SD: Standard deviation
CIs: Confidence intervals.
